# Prevalence of High-Risk Human Papillomaviruses (HPV) in Slovenian Women Attending Organized National Cervical Cancer Screening 14 Years After Implementation of the National HPV Vaccination Program

**DOI:** 10.3390/vaccines13101050

**Published:** 2025-10-13

**Authors:** Mateja Lasič, Anja Oštrbenk, Špela Smrkolj, Klara B. Bohinc, Ana Pflaum, Mario Poljak

**Affiliations:** 1 Department of Gynecology and Obstetrics, Faculty of Medicine, University of Ljubljana, Šlajmerjeva 3, 1000 Ljubljana, Slovenia; mateja.lasic@maurana.com (M.L.); spela.smrkolj@kclj.si (Š.S.); 2 Institute of Microbiology and Immunology, Faculty of Medicine, University of Ljubljana, Zaloška 4, 1000 Ljubljana, Slovenia; anja.ostrbenk@mf.uni-lj.si (A.O.); klara.bohinc@mf.uni-lj.si (K.B.B.); 3 Division of Gynecology and Obstetrics, University Medical Center, Šlajmerjeva 3, 1000 Ljubljana, Slovenia; ana.pflaum@kclj.si

**Keywords:** HPV, prevalence, vaccination, cervical cancer, screening, Slovenia, central and eastern Europe

## Abstract

**Background/Objectives**: To assess overall and type-specific HPV vaccine effectiveness in central and eastern Europe (CEE), the age-stratified prevalence of cervical HPV infection was determined among Slovenian women aged 20 to 64 attending a cervical cancer screening program 14 years after implementation of a national HPV vaccination program, which was then compared with 2009–2010 pre-vaccination data using the same methodological approach. **Methods**: Cervical samples of 4419 women were tested in 2023–2025 using the clinically validated Alinity m HR HPV Assay, and individual HPV types were determined by the Allplex HPV HR Detection assay. Results were compared with 2009–2010 pre-vaccination data generated using the same assay on an age-range matched cohort of women. **Results**: The overall prevalence of the 14 Alinity-targeted HPV types was 10.0% in 2023–2025 versus 13.3% in 2009–2010 (*p* < 0.001). HPV16 prevalence declined from 3.5% to 1.5% (*p* < 0.001), and HPV18 prevalence from 1.1% to 0.5% (*p* = 0.005). In women aged 20 to 24 with 40% uptake of quadrivalent HPV vaccine, overall HPV prevalence dropped from 25.3% to 12.8% (*p* < 0.001). No single case of HPV16/HPV18 infection was detected among vaccinated women. **Conclusions**: The first large-scale, systematic, and methodologically consistent study of HPV vaccine effectiveness in CEE showed a substantial reduction in high-risk HPV prevalence after implementation of the national program, with the greatest decline among women aged 20 to 24, who harbored the highest HPV burden in the pre-vaccination era. These locally acquired data will considerably inform public health strategies on cervical cancer elimination in CEE.

## 1. Introduction

Over 99% of cervical cancers are caused by persistent infection with high-risk human papillomavirus (hrHPV) types [1], making cervical cancer one of the few human malignancies that can be successfully prevented and eventually eliminated through effective public healthcare measures [2]. Achieving this goal relies on a combination of high HPV vaccination uptake, organized cervical cancer screening using a population-based program, and timely treatment of women in whom precancerous lesions are detected [3]. European guidelines recommend HPV testing as the primary screening tool [4], while emphasizing the need to consider local conditions before modifying successful existing screening programs [5]. Therefore, assessing the most recent burden of HPV infection at the population level after implementation of HPV vaccination is essential before modifying or transitioning to HPV-based screening in countries with well-established cytology-based screening programs.

In Slovenia, the organized population-based National Cervical Cancer Screening Program (NCCSP; called ZORA in Slovenian) targets women 20 to 64 years old, who receive conventional Pap cytology screening from gynecologists within a primary healthcare network every 3 years after two consecutive negative screening Pap smears in 12 months upon entering the program. With high screening coverage—72.5% in 3-year intervals (2021–2024) and 86% at 5-year intervals (2019–2024)—the incidence of cervical cancer in Slovenia has substantially decreased since the program’s implementation in 2003. However, it reached a performance plateau and stagnation of incidence decline in the last 10 years, ranging between 8.3 and 12.5 per 100,000 women [6,7]. Furthermore, HPV vaccines have been available in Slovenia since 2006, and the school-based national vaccination program started in the 2009/2010 school year, when free vaccination with three doses of quadrivalent HPV vaccine became available for girls in the sixth grade of primary school (11 to 12 years old). In the 2014/2015 school year, the national HPV vaccination schedule was reduced from three to two doses for those under age 15, and the quadrivalent HPV vaccine was replaced by the nonavalent HPV vaccine in the 2016/2017 school year. Since the 2021/2022 school year, boys 11 to 12 years old have also been included in the national HPV vaccination program. Recently, free catch-up HPV vaccination has been offered to both girls/women and boys/men up to age 26 that missed their initial vaccination opportunity [8,9]. According to data from the Slovenian National Institute of Public Health, HPV vaccination uptake for birth cohorts vaccinated between 2009/2010 and 2015/2016—corresponding to those entering the NCCSP in recent years—ranged from 44.0% to 55.2% [10]. Thus, the current situation (almost equal distribution of non-vaccinated and HPV-vaccinated women with only quadrivalent HPV vaccine in several age cohorts) provides a unique research model for evaluating the effectiveness of HPV vaccination in the age groups carrying the highest burden of HPV infections.

In 2009–2010, the first cross-sectional national study was conducted in Slovenia, enrolling more than 4400 women between 20 and 64 years old attending a routine visit as part of the NCCSP to estimate the pre-vaccination prevalence of cervical HPV infections. In that study, women were screened using traditional Pap cytology and four different clinically validated HPV assays [11,12,13]. In 2009–2010, the overall prevalence of 14 HPV types targeted by the Alinity m HR HPV Assay (Abbott Molecular, Des Plaines, IL, USA)—a clinically validated HPV assay that was also used in the present study—was 13.3%. HPV16 was the most common HPV type, followed by HPV31, HPV51, and HPV52; HPV18 ranked fifth. The overall 14 HPV type prevalence and the prevalence of HPV16 in 2009–2010 was highest among women 20 to 24 years old.

The population impact of HPV vaccination on cervical HPV prevalence has been well documented, with significant reductions in the prevalence of HPV vaccine types being consistently reported from Australia, Canada, the United States [14,15,16,17,18], and western, northern, and southern Europe [19,20,21,22,23,24,25,26,27,28,29,30,31,32,33,34,35,36,37]. In contrast, published data from central and eastern Europe remain limited and of poor evidence quality. The present study is the largest study in the region aiming to assess HPV vaccine effectiveness and the further benefits of HPV vaccination at the population level (e.g., herd immunity, cross-protection, and type replacement), and is the first with a methodologically consistent comparison of HPV prevalence before and after HPV vaccine implementation. To achieve the study aims, we determined the overall and type-specific age-stratified prevalence of cervical hrHPV infection in a representative cohort of over 4400 Slovenian women aged 20 to 64 years old attending an organized national cervical cancer screening program 14 years after the implementation of a national HPV vaccination program, which was compared with pre-vaccination data obtained from 2009 to 2010 using the same methodological approach.

## 2. Materials and Methods

### 2.1. Study Design

We conducted a cross-sectional prospective study, enrolling women 20 to 64 years old that attended a routine gynecological examination as part of the organized NCCSP between December 2023 and March 2025 at 21 outpatient clinics across various Slovenian regions. Only women with a normal cytology result from the previous screening round were eligible, as well as those with a first cervical smear collection upon entry into the screening program. Exclusion criteria included pregnancy, menstrual bleeding, colpitis and/or cervicitis, and other medical conditions that would prevent adequate Pap smear collection. Women that had undergone a hysterectomy or were undergoing a follow-up cervical smear after abnormal cytological findings, treatment for cervical pathology, or suspicion of malignancy were also excluded. Written individual informed consent was obtained from all subjects involved in the study. The study was conducted in accordance with the Declaration of Helsinki, and it was approved by the National Medical Ethics Committee of the Republic of Slovenia (consent number 0120-218/2023/10).

### 2.2. Sample and Data Collection

Cervical samples for HPV testing were collected during the gynecological examination following a routine conventional Pap smear as part of the NCCSP procedure and placed into ThinPrep PreservCyt Solution (Hologic Inc., Marlborough, MA, USA). The gynecologist completed a detailed data collection form, including information on the participant’s HPV vaccination status. Participants also completed an anonymous questionnaire that included self-reported data on HPV vaccination status. Data collection forms, anonymous questionnaires, and cervical smear samples for HPV testing were labeled with a unique anonymous participant code.

### 2.3. HPV Testing

All specimens were tested using the Alinity m HR HPV Assay (Alinity), a clinically validated HPV assay that was run on the automated Alinity m system, following the manufacturer’s instructions [38]. The Alinity assay is a qualitative real-time PCR-based assay that targets 14 HPV types and allows individual identification of HPV16, HPV18, and HPV45, and concurrent detection of other HPV types in two groups: group A (HPV31, 33, 52, and 58) and group B (HPV35, 39, 51, 56, 59, 66, and 68), at clinically relevant infection levels. All samples positive by Alinity were further tested with the clinically validated Allplex HPV HR Detection assay (Allplex; Seegene Inc., Seoul, Republic of Korea), following the manufacturer’s instructions, as previously described [39]. Allplex is a qualitative multiplex real-time PCR-based assay that allows simultaneous amplification and individual detection of 14 HPV types (HPV16, 18, 31, 33, 35, 39, 45, 51, 52, 56, 58, 59, 66, and 68) as well as the beta-globin gene as an internal control.

### 2.4. Data Analysis

We compared data from 2023 to 2025 with pre-vaccination data obtained in a cross-sectional study with the same design conducted in 2009–2010. Both cohorts (before and after implementation of HPV vaccination) included Slovenian women aged 20 to 64 years old that had undergone routine gynecological examinations within the NCCSP, applying identical inclusion and exclusion criteria. For both cohorts, HPV prevalence was estimated using the same clinically validated HPV assay (Alinity), allowing for direct and methodologically consistent comparisons between the two time points.

Using descriptive statistics, we first estimated the overall age-stratified prevalence of cervical infections with 14 HPV types targeted by Alinity (HPV16, 18, 31, 33, 35, 39, 45, 51, 52, 56, 58, 59, 66, and 68). In addition, we assessed the type-specific age-stratified prevalence for 12 hrHPV types (HPV16, 18, 31, 33, 35, 39, 45, 51, 52, 56, 58, and 59) recently classified by the International Agency for Research on Cancer (IARC) as Group 1 “carcinogenic to humans” [40]. In this last classification, the IARC omitted HPV66 and HPV68 from Group 1 “carcinogenic to humans,” leaving 12 hrHPV types (hereafter 12 IARC hrHPV types) because detection of HPV66 and HPV68 rarely identifies women with clinically significant disease but is responsible for substantial overtreatment. We calculated the overall prevalence of 14 HPV targeted by Alinity and type-specific 12 IARC hrHPV prevalence for women aged 20 to 64 years old in the pre-vaccination cohort in 2009–2010 and in the cohort after HPV vaccination implementation in 2023–2025, with 95% confidence intervals (CI) using the Wilson CI for a proportion. In addition, we assessed both overall and type-specific hrHPV prevalence among women that were age-eligible for HPV vaccination during adolescence and were 20 to 24 years old at the time of study recruitment in 2023–2025. Prevalence estimates were stratified by vaccination status. To quantify differences between groups, we calculated the relative reduction and risk ratio (RR), whereby relative reduction was defined as 1 − RR. Risk ratios were computed either as RR = P_2023–2025_/P_2009–2010_ for temporal comparisons or as RR = P_vacc_/P_non-vacc_ for comparisons by vaccination status, with P representing HPV prevalence in the respective group. In accordance with the recommendations of the World Health Organization Strategic Advisory Group of Experts on Immunization (SAGE), participants were classified as vaccinated if they had received at least one dose of the HPV vaccine before age 15, provided that vaccination status was concordantly reported by both the woman and her gynecologist. Differences in prevalence were tested with Pearson’s chi-square or Fisher’s exact test (for small samples). A two-tailed *p*-value < 0.05 indicated statistical significance. Data were analyzed using IBM SPSS Statistics, version 30 (IBM Corp., Armonk, NY, USA) and R software version 4.5.1 (Free Software Foundation, Boston, MA, USA).

## 3. Results

A total of 4429 eligible women were invited to participate in this study, of whom eight were excluded due to a missing ThinPrep sample or sample spillage during transport and two withdrew consent. Thus, data from a total of 4419 participants were included in the final analysis.

**Figure 1:**
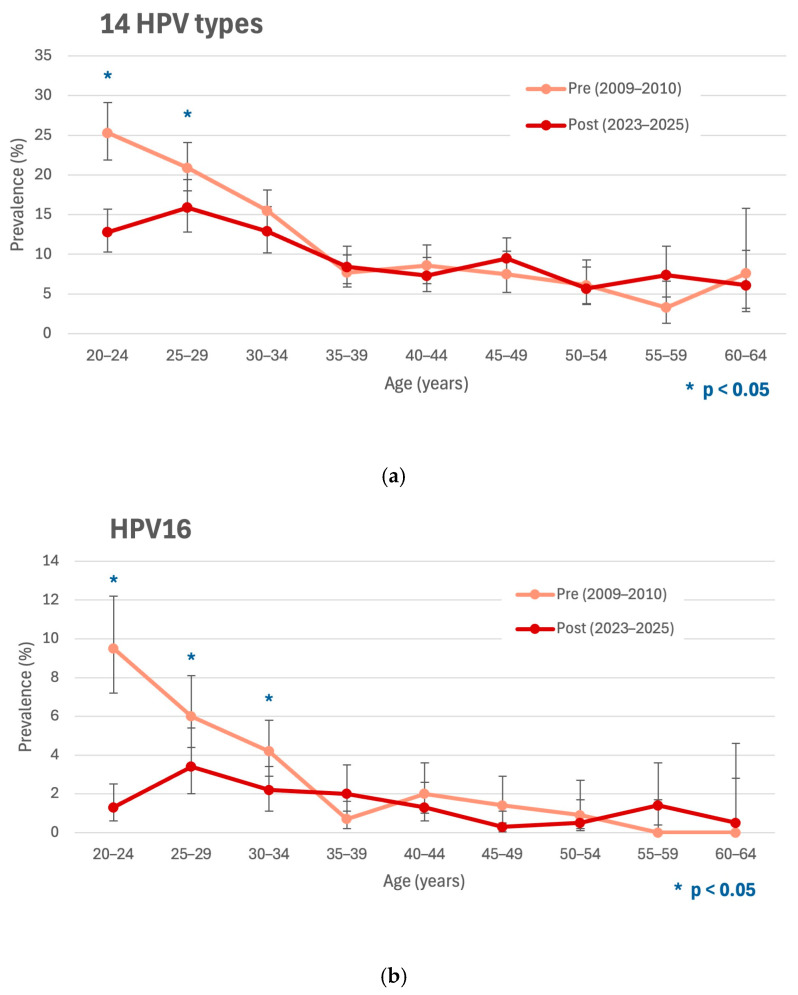

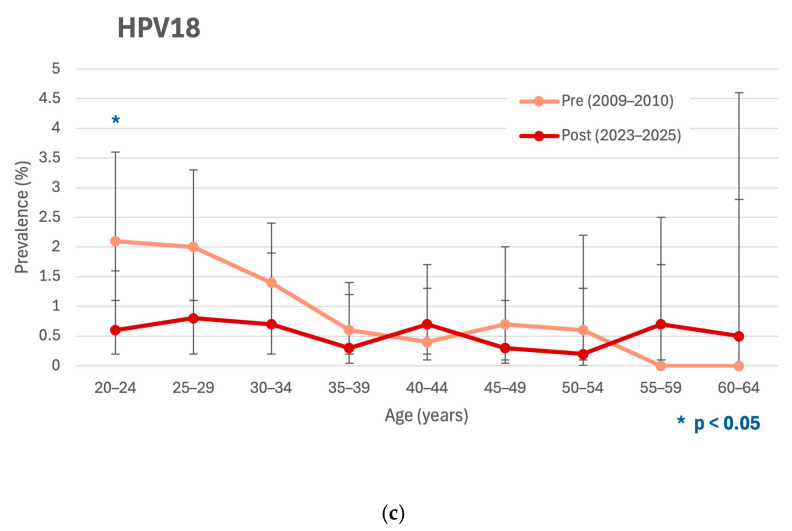
Comparison of pre- and post-vaccination prevalence of cervical infection with 14 HPV types (HPV16, 18, 31, 33, 35, 39, 45, 51, 52, 56, 58, 59, 66, and 68) targeted by the Alinity m HR HPV Assay, HPV16 and HPV18 infection with 95% confidence intervals stratified by age among Slovenian women attending organized national cervical cancer screening in 2009–2010 and 2023–2025, respectively. According to data from the Slovenian National Institute of Public Health, HPV vaccination uptake for birth cohorts vaccinated between 2009/2010 and 2015/2016—corresponding to those 20 to 27 years old at the time of the present study—ranged from 44.0% to 55.2%. Other age groups had a negligible level of HPV vaccination coverage and were vaccinated outside the national vaccination program (mainly at age 20 or older). Statistically significant *p*-values are indicated with an asterisk (*). (**a**) Overall prevalence of 14 HPV types targeted by the Alinity m HR HPV Assay in 2009–2010 and 2023–2025. (**b**) Prevalence of HPV16 as determined by full genotyping assay in 2009–2010 and 2023–2025. (**c**) Prevalence of HPV18 as determined by full genotyping assay in 2009–2010 and 2023–2025.

The overall prevalence of 14 HPV types targeted by Alinity in 2023–2025 among Slovenian women aged 20 to 64 years old was 10.0% (95% CI: 9.2–10.9%). Compared to the pre-vaccination prevalence in 2009–2010 of 13.3% (95% CI: 12.3–14.3%), the difference was significant (*p* < 0.001). Among women aged 20 to 24 years old, the overall prevalence of 14 HPV types targeted by Alinity declined significantly from 25.3% (95% CI: 22.0–29.0%) in 2009–2010 to 12.8% (95% CI: 10.4–15.6%) in 2023–2025 (*p* < 0.001). As shown in Figure 1, the highest prevalence of 14 HPV types in 2023–2025 was observed in the 25 to 29 age group at 15.9% (95% CI: 12.8–19.4%); however, this was still significantly lower than the pre-vaccination prevalence, estimated in 2009–2010 at 20.9% (95% CI: 18.0–24.1%; *p* = 0.026). An additional peak in the prevalence of 14 HPV types in 2023–2025 was noted in the 45 to 49 age group, seemingly shifting from the 40 to 44 age group compared to data from 2009 to 2010 (Figure 1).

The type-specific prevalence of 12 IARC hrHPV types was assessed for the total study population (women 20 to 64 years old; Table 1) and separately for the 20 to 24 age group (Table 2). In the latter, a significant reduction in HPV16 and HPV18 prevalence was observed in 2023–2025 compared to 2009–2010, from 9.5% (95% CI: 7.4–12.1%) to 1.3% (95% CI: 0.6–2.5%) for HPV16 (*p* < 0.001) and from 2.1% (95% CI:1.2–3.6%) to 0.6% (95% CI: 0.2–1.6%) for HPV18 (*p* = 0.041), corresponding to a relative reduction of 86.7% (95% CI: 72.2–93.6%) and 69.4% (95% CI: 5.7–90.1%), respectively (Table 2). A significant decline in HPV16 prevalence was observed in the 25 to 29 age group as well (Figure 1). In the vaccination-eligible birth cohorts aged 20 to 24 years old in 2023–2025, the most prevalent types were HPV51, HPV56, and HPV59 (each 2.4%), followed by HPV52 (1.7%) and HPV39 (1.4%). The two most prevalent HPV types in 2009–2010, HPV16 and HPV31, ranked sixth, and HPV18 ranked ninth in 2023–2025. As shown in Table 1, when considering all age groups combined, significant type-specific prevalence reductions in 2023–2025 compared to 2009–2010 were also observed for HPV31 (*p* < 0.001), HPV45 (*p* = 0.027), HPV51 (*p* = 0.004), HPV52 (*p* = 0.027), and HPV56 (*p* = 0.005). Simultaneous infections with two or more of the 12 IARC hrHPV types (hereafter multiple infections) were detected in 2023–2025 in 17.2% (76/443) of women, significantly lower than the 22.8% (134/589) observed in the pre-vaccination cohort in 2009–2010 (*p* < 0.001). The most pronounced decrease in the prevalence of multiple infection was seen in the 20 to 24 age group (Table 2).

In HPV vaccination-eligible birth cohorts aged 20 to 24 years old at the time of this study, 40.0% (253/632) of women had received at least one dose of the quadrivalent HPV vaccine before age 15. When stratified by HPV vaccination status, no single case of HPV16 and HPV18 infection was detected among vaccinated women, compared to 2.1% and 1.1%, respectively, in their unvaccinated peers (Table 3). The most common HPV types detected among HPV-vaccinated women in this age group were HPV59 (3.2%) and HPV51 (2.8%), whereas HPV56 (2.4%), HPV16 (2.1%), and HPV51 (2.1%) were most prevalent among unvaccinated women. A modestly higher, yet statistically non-significant, prevalence of non-HPV16/18 types (HPV39, 51, 52, 58, and 59) was observed among vaccinated women compared to non-vaccinated women (Table 3). This contributed to a marginally increased, but still statistically non-significant, overall prevalence of any of the 12 IARC hrHPV types (RR = 1.13, 95% CI: 0.7–1.8%) among vaccinated women (Table 3).

## 4. Discussion

In the present study, we assessed the real-time impact of HPV vaccination at the population level in Slovenia after the first seven age cohorts of Slovenian women that were offered the quadrivalent HPV vaccine at ages 11 to 12 (from the 2009/10 to 2015/16 school years) entered the NCCSP (they are now 20 to 27 years old). We observed a significant reduction in the overall prevalence of 14 HPV types targeted by Alinity, from 13.3% before the implementation of school-based national HPV vaccination to 10.0% afterward. This decline was particularly marked for HPV16, which dropped from 3.5% to 1.5%, and HPV18, from 1.1% to 0.5%, with the most pronounced decline in the 20 to 24 age group—historically the group with the highest hrHPV burden prior to vaccine introduction. Notably, not a single case of HPV16 and HPV18 infection was detected among vaccinated women in this age group, supporting the strong elimination potential of HPV vaccines for these two clinically most oncogenic HPV types, which cause more than 70% of cervical cancers worldwide.

Studies from Australia, Canada, and the United States demonstrated a substantial decline in the prevalence of vaccine-targeted HPV types following the introduction of HPV vaccination programs. In Australia, the prevalence of HPV6, 11, 16, and 18 among women aged 18 to 24 years old dropped from 29% to 7% (*p* < 0.0001), with an adjusted vaccine effectiveness of 86% for vaccine-targeted types and 58% for related non-vaccine types [14]. Similarly, a Canadian study found a markedly lower prevalence of these types in quadrivalent HPV-vaccinated versus unvaccinated women (1.5% vs. 11.0%); with no significant changes in the prevalence of other HPV types [17]. In the United States, the prevalence of vaccine-targeted HPV types decreased from 11.5% to 4.3% among females 14 to 19 years old and from 18.5% to 12.1% among those aged 20 to 24 years old [18].

Similar observations on the impact of HPV vaccination on the prevalence of cervical HPV infection were reported in several European studies [19,20,21,22,23,24,25,26,27,28,29,30,31,32,33,34,35,36,37]. A Scottish study observed a statistically significant reduction in HPV16/18 prevalence among vaccinated women with a prevalence of 11.0% compared to 29.4% in unvaccinated women (*p* < 0.0001) [19]. A subsequent larger Scottish study confirmed these findings, reporting that HPV16/18 prevalence among vaccinated women decreased to 4.5%, corresponding to a vaccine effectiveness of 89.1% [20]. In England, HPV16/18 prevalence in sexually active females aged 16 to 18 years old offered vaccination at age 12 to 13 was less than 1%, compared to over 15% before the introduction of HPV vaccination (*p* < 0.001) [21]. In Belgium, two large studies have demonstrated significant post-vaccination effects. The first study, conducted in 2015, reported an early decline in HPV16 prevalence among the youngest age group (15–19 years), with a relative risk of 0.61 [23]. A more recent Belgian study from 2023 confirmed a significant reduction in HPV16/18 prevalence among women aged 20 to 23 years old [24]. A Danish population-based study showed a 95% reduction in HPV16/18 prevalence among 23-year-olds [25], supported by a large Nordic study showing a significant decline of HPV6, 11, 16, and 18 infections among women aged 18 to 26 years old (*p* < 0.001) [26]. Other western European countries such as Germany, France, and the Netherlands have also reported similar outcomes, showing significant reductions in the prevalence of vaccine types and high vaccine effectiveness [27,28,29,30,31,32,33]. In southern Europe, studies from Italy, Spain, and Portugal also demonstrated a strong vaccine impact, with vaccine effectiveness reaching up to 95% in the youngest cohorts [34,35,36,37].

In contrast to other parts of Europe, data from central and eastern Europe remain scarce and of suboptimal quality, making direct comparison with data generated in western and northern Europe challenging. To date, only two studies from the region have reported on HPV prevalence after the introduction of HPV vaccination. In Bulgaria, a comparison of data from 2012 and 2017 showed only a minimal decline in HPV16 prevalence (from 46.0% to 45.6%), which the authors attributed to low vaccine uptake and the lack of an organized national vaccination program [41]. Similarly, a Croatian study reported a non-significant reduction in HPV16/18 prevalence among young women; however, its small sample size (*n* = 321) limited statistical power and generalizability [42]. Although these two studies were among the first to contribute data on HPV prevalence in the region in the HPV vaccination era, they lacked a consistent methodology and systematic comparison of pre- and post-vaccination data, limiting their ability to fully assess HPV vaccine impact. Thus, to the best of our knowledge this study is the first and largest study to date to assess HPV vaccination effectiveness in central and eastern Europe, where HPV vaccination uptake is much lower than in northern and western Europe [43] and where cervical cancer incidence and mortality are substantially higher [44,45].

A concurrent decline in HPV16/18 prevalence observed in our study also among unvaccinated women after the implementation of the school-based national immunization program confirmed that HPV vaccination can offer indirect protection through herd immunity, in line with previously published data from some European countries [19,20,21,27,32]. In addition to a striking reduction in HPV16 /18 prevalence in the 20 to 24 age group, we also observed a reduction in hrHPV prevalence in the 25 to 29 age group, likely due to the inclusion of three birth cohorts that had been eligible for HPV vaccination during adolescence through the national vaccination program. In addition, we observed a significant decline in the prevalence of HPV31, 45, 52, and 58 in women aged 20 to 24 years old, suggesting cross-protection and further enhancing the similar evidence from previous studies [19,20,21,29,31,32].

In many countries, accurate assessment of the HPV vaccine population impact is partially limited by the lack of individual-level vaccination records and their linkage with screening as well as cancer registries. Similarly, in Slovenia, HPV vaccination coverage is monitored at the regional and national levels by the Slovenian National Institute of Public Health, but individual-level vaccination records are still not linked to individual screening and clinical records, and individual-level vaccination data are not routinely reported in any of the national registers. Furthermore, regional variations in HPV vaccine uptake in Slovenia remain substantial [8,9,10]. In our study, HPV vaccination status was therefore assigned only to women for whom concordant self-reported and gynecological data were available, resulting in an observed HPV vaccination uptake rate of 40.0% (253/632; Table 3) in the 20 to 24 age group. According to official national data, the average coverage in vaccine-eligible birth cohorts was approximately 50% [10]. It is therefore plausible that some women participating in our study were misclassified as unvaccinated even though they had actually received the HPV vaccine. Although such misclassification could contribute to a slightly elevated HPV16/18 prevalence in the unvaccinated group, the overall number of HPV16/18-positive cases was very low, and the strong effect of herd immunity would likely mitigate any potential bias in the prevalence estimates.

In HPV vaccination-eligible birth cohorts, now aged 20 to 24 years old, an unexpected slightly higher—but statistically non-significant—prevalence of non-HPV16/18 types was observed among vaccinated women, suggesting a modest non-significant increased risk of infection with non-HPV16/18 types compared to unvaccinated peers [46]. However, as discussed above, potential misclassification of vaccination status—combined with the small overall number of HPV-positive cases—may have influenced this pattern. Some women assigned as unvaccinated were likely vaccinated, potentially leading to a slight overestimation of prevalence in the vaccinated group. Importantly, the overall post-vaccination prevalence of non-HPV16/18 types in this age group was lower than in the pre-vaccination cohort. Taken together, these findings do not support the occurrence of type replacement following vaccination.

## 5. Conclusions

The main strength of our study lies in its large nationally representative sample of women attending organized national cervical screening, with high 3-year and 5-year screening coverage making it the largest and the first post-vaccination HPV prevalence study in a representative cervical screening population in central and eastern Europe to date. It is also the first study providing a methodologically consistent comparison of HPV type-specific prevalence before and after implementation of an organized national HPV vaccination program in this region, since the same clinically validated HPV assay was used for HPV prevalence assessment in both cohorts enrolled 14 years apart. An additional strength of the study, despite some limitations, is that all participants had double verified vaccination status at the individual level, which was more the exception than the rule in similar prevalence studies performed in the western world.

There are two key points of significance of our findings: national and regional. At the national level, Slovenia continues to use conventional cytology (Pap smear) as the primary cervical cancer screening method, performed by gynecologists in outpatient settings at 3-year intervals for women aged 20 to 64 years old. In this context, our results provide crucial further evidence to support, guide, and facilitate the transition to HPV-based primary screening in the country, aligning with international and European screening recommendations and improving screening effectiveness in the HPV vaccination era. A thorough monitoring of HPV prevalence in the vaccination era as well as behavior at the population level is essential for anticipating epidemiological trends and tailoring preventive strategies. This is especially relevant in Slovenia, where HPV vaccination uptake remains relatively low, varies substantially by region, and has been affected by broader social factors such as rising vaccine hesitancy and the impact of the COVID-19 pandemic. These challenges highlight the need for continuous monitoring of HPV epidemiology to inform national policies on immunization and screening. Our study provides important evidence that may contribute to stronger public health efforts and serve as a valuable incentive to support the transition to primary HPV-based cervical cancer screening in Slovenia, as well as to increase currently suboptimal HPV vaccination uptake across the country.

In addition to its national contribution, this study is particularly relevant for central and eastern Europe, where many countries have historically relied on suboptimal cervical cancer screening practices and have only recently implemented HPV vaccination programs with low and, in the best case, modest vaccine uptake [47]. As a result, the cervical cancer incidence and mortality in the region remain among the highest in the world, underscoring the urgent need for locally acquired data to inform future public health strategies. Given the comparable distribution of HPV types and shared healthcare system challenges across the region, our findings provide important evidence that can support and inform coordinated efforts toward cervical cancer control and, hopefully, its subsequent elimination in central and eastern Europe.

## Figures and Tables

**Table 1:** Overall prevalence of infection with 14 Alinity HPV types and type-specific prevalence of 12 IARC hrHPV types among Slovenian women aged 20 to 64 years old before and 14 years after the implementation of the national HPV vaccination program with 44.0% to 55.2% vaccination uptake among those aged 20 to 27 years old at the time of the study. Other age groups had a negligible level of HPV vaccination uptake. HPV prevalence was assessed using the same clinically validated HPV assay (the Alinity m HR HPV Assay) in 2009–2010 and 2023–2025. All Alinity m HR HPV Assay–positive samples were further tested with the clinically validated Allplex HPV HR Detection assay to detect individual HPV type(s).

HPV Type	Pre-Vaccination Period (2009–2010), Women Age 20–64 (*n* = 4425)			After Implementation of HPV Vaccination (2023–2025), Women Age 20–64 (*n =* 4419)			*p*-Value *	Relative Reduction (%)	95% CI
	*n*	Prevalence (%)	95% CI	*n*	Prevalence (%)	95% CI			
HPV positive ^a^	589	13.3	12.3–14.3	443	10.0	9.2–10.9	**<0.001**	24.7	15.4–32.9
Multiple HPV types ^b^	134	3.0	2.6–3.6	76	1.7	1.4–2.1	**<0.001**	43.2	25.0–57.0
HPV 16/18 ^c^	199	4.5	3.9–5.1	87	2.0	1.6–2.4	**<0.001**	56.2	43.9–65.9
HPV16	157	3.5	3.0–4.1	66	1.5	1.2–1.9	**<0.001**	57.9	44.1–68.3
HPV18	48	1.1	0.8–1.4	24	0.5	0.4–0.8	**0.005**	49.9	18.4–69.3
HPV31	114	2.6	2.1–3.1	63	1.4	1.1–1.8	**<0.001**	44.7	24.9–59.2
HPV33	32	0.7	0.5–1.0	23	0.5	0.3–0.8	0.225	28.0	−22.8–57.8
HPV35	9	0.2	0.1–0.4	19	0.4	0.3–0.7	0.058	−111.4	−366.7–4.3
HPV39	50	1.1	0.9–1.5	39	0.9	0.6–1.2	0.244	21.9	−18.5–48.5
HPV45	42	0.9	0.7–1.3	24	0.5	0.4–0.8	**0.027**	42.8	5.7–65.3
HPV51	81	1.8	1.5–2.3	48	1.1	0.8–1.4	**0.004**	40.7	15.4–58.4
HPV52	77	1.7	1.4–2.2	52	1.2	0.9–1.5	**0.027**	32.4	4.1–52.3
HPV56	31	0.7	0.5–1.0	57	1.3	1.0–1.7	**0.005**	−84.1	−184.6–19.1
HPV58	29	0.7	0.5–0.9	24	0.5	0.4–0.8	0.494	17.1	−42.1–51.7
HPV59	48	1.1	0.8–1.4	45	1.0	0.8–1.4	0.759	6.1	−40.7–37.4

Abbreviations: *n* = number of women; CI = confidence interval. ^a^ Women with an HPV-positive Alinity m HR HPV Assay result. ^b^ Women with simultaneous infection with two or more 12 IARC hrHPV types. ^c^ Women with infection with either HPV16 and/or HPV18 or both. * Statistically significant *p*-values are shown in bold.

**Table 2:** Overall prevalence with 14 Alinity HPV types and type-specific prevalence of 12 IARC hrHPV types among Slovenian women aged 20 to 24 years old before and 14 years after the implementation of the national HPV vaccination program with 45.5% to 55.2% vaccination uptake in this age group. HPV prevalence was assessed using the same clinically validated HPV assay (the Alinity m HR HPV Assay) in 2009–2010 and 2023–2025. All Alinity m HR HPV Assay–positive samples were further tested with the clinically validated Allplex HPV HR Detection assay to detect individual HPV type(s).

HPV Type	Pre-Vaccination Period (2009–2010), Women Age 20–24 *(n =* 580)			After Implementation of HPV Vaccination (2023–2025), Women Age 20–24 *(n* = 632)			*p*-Value *	Relative Reduction (%)	95% CI
	*n*	Prevalence (%)	95% CI	*n*	Prevalence (%)	95% CI			
HPV positive ^a^	147	25.3	22.0–29.0	81	12.8	10.4–15.6	**<0.001**	49.4	35.3–60.5
Multiple HPV types ^b^	52	9.0	6.9–11.6	21	3.3	2.2–5.0	**<0.001**	62.9	39.3–77.4
HPV 16/18 ^c^	64	11.0	8.7–13.8	12	1.9	1.1–3.3	**<0.001**	82.8	68.5–90.6
HPV16	55	9.5	7.4–12.1	8	1.3	0.6–2.5	**<0.001**	86.7	72.2–93.6
HPV18	12	2.1	1.2–3.6	4	0.6	0.2–1.6	**0.041**	69.4	5.7–90.1
HPV31	28	4.8	3.4–6.9	8	1.3	0.6–2.5	**<0.001**	73.8	42.9–88.0
HPV33	7	1.2	0.6–2.5	6	0.9	0.4–2.1	0.783	21.3	−132.7–73.4
HPV35	2	0.3	0.1–1.2	3	0.5	0.2–1.4	1.000	−37.7	−720.9–76.9
HPV39	16	2.8	1.7–4.4	9	1.4	0.8–2.7	0.110	48.4	−15.9–77.0
HPV45	13	2.2	1.3–3.8	2	0.3	0.1–1.1	**0.003**	85.9	37.7–96.8
HPV51	25	4.3	2.9–6.3	15	2.4	1.4–3.9	0.059	44.9	−3.4–70.7
HPV52	25	4.3	2.9–6.3	11	1.7	1.0–3.1	**0.008**	59.6	18.7–80.0
HPV56	13	2.2	1.3–3.8	15	2.4	1.4–3.9	0.879	−5.9	−120.6–49.2
HPV58	12	2.1	1.2–3.6	4	0.6	0.2–1.6	**0.041**	69.4	5.7–90.1
HPV59	15	2.6	1.6–4.2	15	2.4	1.4–3.9	0.812	8.2	−86.1–54.7

Abbreviations: *n* = number of women; CI = confidence interval. ^a^ Women with an HPV-positive Alinity m HR HPV Assay result. ^b^ Women with simultaneous infection with two or more 12 IARC hrHPV types. ^c^ Women with infection with either HPV16 and/or HPV18 or both. * Statistically significant *p*-values are shown in bold.

**Table 3:** Overall prevalence and type-specific prevalence of 12 IARC hrHPV types among Slovenian women aged 20 to 24 years old before and 14 years after the implementation of the national HPV vaccination program with 45.5% to 55.2% vaccination uptake in this age group, stratified by vaccination status. HPV prevalence was assessed using a clinically validated HPV assay (the Alinity m HR HPV Assay), and all positive samples were further tested with the clinically validated Allplex HPV HR Detection assay to detect individual HPV type(s).

HPV Type	Vaccinated *(n* = 253)			Unvaccinated *(n* = 379)			*p*-Value *	Risk Ratio (RR) **	95% CI
	*n*	Prevalence (%)	95% CI	*n*	Prevalence (%)	95% CI			
12hr HPV positive	31	12.3	8.8–16.9	41	10.8	8.1–14.3	0.578	1.13	0.7–1.8
Multiple 12 hrHPV ^a^	8	3.2	1.6–6.1	13	3.4	2.0–5.8	0.854	0.92	0.4–2.2
HPV16/18 ^b^	0	0.0	0.0–1.5	12	3.3	1.8–5.5	**0.002**	0.06	0.0–1.0
HPV16	0	0.0	0.0–1.5	8	2.1	1.1–4.1	**0.024**	0.09	0.0–1.5
HPV18	0	0.0	0.0–1.5	4	1.1	0.4–2.7	0.154	0.17	0.0–3.1
HPV31	3	1.2	0.4–3.4	5	1.3	0.6–3.1	1.000	0.90	0.2–3.7
HPV33	2	0.8	0.2–2.8	4	1.1	0.4–2.7	1.000	0.75	0.1–4.1
HPV35	1	0.4	0.1–2.2	2	0.5	0.1–1.9	1.000	0.75	0.1–8.2
HPV39	6	2.4	1.1–5.1	3	0.8	0.3–2.3	0.167	3.00	0.8–11.9
HPV45	0	0.0	0.0–1.5	2	0.5	0.1–1.9	0.519	0.30	0.1–6.2
HPV51	7	2.8	1.3–5.6	8	2.1	1.1–4.1	0.604	1.31	0.5–3.6
HPV52	5	2.0	0.8–4.5	6	1.6	0.7–3.4	0.762	1.25	0.4–4.0
HPV56	6	2.4	1.1–5.1	9	2.4	1.3–4.5	1.000	1.00	0.4–2.8
HPV58	2	0.8	0.2–2.8	2	0.5	0.1–1.9	1.000	1.50	0.2–10.6
HPV59	8	3.2	1.6–6.1	7	1.8	0.9–3.8	0.287	1.71	0.6–4.7

Abbreviations: hrHPV = high-risk HPV types*; n* = number of women; CI = confidence interval. ^a^ Women with simultaneous infection with two or more 12 IARC hrHPV types. ^b^ Women with single infection either with HPV16 and/or HPV18 or both. * Statistically significant *p*-values are shown in bold. ** If no women with infection were identified in specific groups, a Haldane–Anscombe correction was implied.

## Data Availability

Anonymized datasets will be made available from the corresponding author upon reasonable request.

## Abbreviations

The following abbreviations are used in this manuscript:

HPV	Human papillomavirus
hrHPV	High-risk human papillomavirus
NCCSP	National cervical cancer screening program
IARC	International Agency for Research on Cancer
CI	Confidence interval
RR	Risk ratio
